# EpiAlignment: alignment with both DNA sequence and epigenomic data

**DOI:** 10.1093/nar/gkz426

**Published:** 2019-05-22

**Authors:** Jia Lu, Xiaoyi Cao, Sheng Zhong

**Affiliations:** Department of Bioengineering, University of California San Diego, La Jolla, CA 92093, USA

## Abstract

Comparative epigenomics, which subjects both epigenome and genome to interspecies comparison, has become a powerful approach to reveal regulatory features of the genome. Thus elucidated regulatory features surpass the information derived from comparison of genomic sequences alone. Here, we present EpiAlignment, a web-based tool to align genomic regions with both DNA sequence and epigenomic data. EpiAlignment takes DNA sequence and epigenomic profiles derived by ChIP-seq from two species as input data, and outputs the best semi-global alignments. These alignments are based on EpiAlignment scores, computed by a dynamic programming algorithm that accounts for both sequence alignment and epigenome similarity. For timely response, the EpiAlignment web server automatically initiates up to 140 computing threads depending on the size of user input data. For users’ convenience, we have pre-compiled the comparable human and mouse epigenome datasets in matched cell types and tissues from the Roadmap Epigenomics and ENCODE consortia. Users can either upload their own data or select pre-compiled datasets as inputs for EpiAlignment analyses. Results are presented in graphical and tabular formats where the entries can be interactively expanded to visualize additional features of these aligned regions. EpiAlignment is available at https://epialign.ucsd.edu/.

## INTRODUCTION

Epigenomic modifications contribute to the implementation of genomic functions in defining cell identity, coordinating organismal development (1,2), and regulating personal cognition and behavior (3,4). A set of recent efforts have established the proof of principle that comparative analyses of interspecies epigenomes can lead to functional annotation of non-coding regulatory genomic sequences (5,6). These regulatory sequences could not be discovered by sequence comparison alone, due to obscure sequence conservation that is likely a result of intricate interplays of negative and positive selections on nested sequence segments (7,8). Subsequent studies have clarified the evolutionary properties of primate (9,10), vertebrate (11) and teleost epigenomes (12) and have revealed co-evolution properties of the genomes and the epigenomes (13–16). Specialized probabilistic models (17) and computational tools (18,19) have been developed for comparative epigenomic analyses. The most recent efforts have expanded interspecies comparisons to incorporate the 3D organization of the genome (20–22). These efforts highlight how comparative epigenomics, an emerging field, leverages evolutionary patterns of epigenomes to functionally annotate genomes. The rapid growth of functional genomic data also provides ample resources for comparative studies. To date, major epigenome consortia including the RoadMap Epigenomics Project (23) and the ENCODE Project (24,25) have produced thousands of high-throughput functional genomic datasets in more than 80 tissue types of several species. Given the advances in analytical methods and the explosive growth of epigenomic data, a computational tool for the integrative comparison of the genome and the epigenome is in demand.

Here we present the EpiAlignment web server, a pairwise alignment tool for both the genome and the epigenome. EpiAlignment aligns two genomic regions from two species based on their DNA sequences and epigenomic modifications. The web server provides a database of pairwise ChIP-seq datasets for users’ convenience, which contains peak files of 56 human and 70 mouse ChIP-seq experiments from 15 matched tissue types and cell lines. Users can either upload their own data or select pre-compiled datasets as inputs for EpiAlignment analyses.

**Figure 1.**
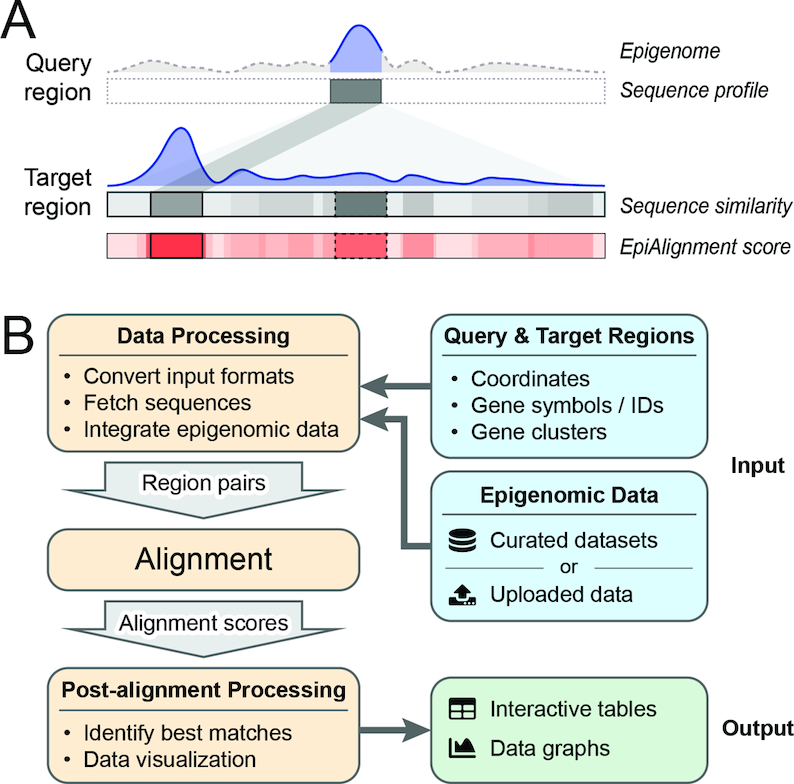
Overview of EpiAlignment. (**A**) Demonstration of the default alignment mode. The query region (up, dark gray box) and the target region (down, gray bar) are provided by the user as inputs, together with the epigenomic data (purple). The sequence similarity to the query region varies along the target region (grayscale in the ‘sequence similarity’ bar). Within the target region, the gray box with dotted border represents the best sequence match with the highest sequence similarity, and the gray box with solid border represents a sub-region with moderate sequence similarity. In the analysis, EpiAlignment yields scores accounting for both sequence and epigenomic similarities (shades of red in the bottom bar), revealing the best match (red box with solid border) with overall similarity over the best sequence match (red box with dotted border). (**B**) The EpiAlignment workflow.

EpiAlignment supports two alignment modes: the one-vs-one mode (default) and the many-vs-many mode, corresponding to two types of analyses. In the one-vs-one mode, the user-defined genomic region from one species (query region) is aligned to the user-defined genomic region in the other species (target region) to identify a continuous subset of the target region with the best similarity to the query region. The similarity is evaluated based on both genomic and epigenomic data in the query and the target regions (Figure 1A). The many-vs-many mode aligns each query region in one species against all the user-defined target regions in the other species to find the best match (Supplementary Figure S1). The EpiAlignment scores and best matches are reported in graphical and tabular formats (Figure 1B). The aligned regions can be further visualized in the UCSC genome browser (26) and the Genomic Interaction Visualization Engine (GIVE) (19) by following links provided in the result tables.

## METHODS AND IMPLEMENTATION

### EpiAlignment algorithm

The EpiAlignment algorithm aims to identify the optimal alignment between two genomic regions, denoted by *A* and *B*, with information from both the genome and the epigenome. Each genomic region contains two types of data, namely the DNA sequence and the epigenomic profile, denoted by ${A_g},\ {B_g}$ and ${A_e},\ {B_e}$ respectively. Thus $A\ = \ \{ {{A_g},\ {A_e}} \}$, and $B\ = \ \{ {{B_g},\ {B_e}} \}$. ${A_e}$ and ${B_e}$ consist of epigenomic states on the underlying DNA bases, which are represented by binary states (1,0). The epigenomic state equals to 1 within regions marked by epigenomic modifications and 0 outside such regions (Supplementary Figure S2).

We denote an alignment between the two genomic regions by *α*. The following target function is employed to evaluate the quality of *α*:

\begin{eqnarray*}T({\alpha, A, B}) &=& T\left({\alpha, ({{A_g}, {A_e}}), ({{B_g}, {B_e}})}\right)\nonumber\\ &&= S({\alpha, {A_g}, {B_g}}) + wE({\alpha, {A_e}, {B_e}})\end{eqnarray*}

where $T( {\alpha ,\ A,\ B} )$ is the final EpiAlignment score, $S( {\alpha ,\ {A_g},\ {B_g}} )$ is the sequence alignment score of the two sequences, $E( {\alpha ,\ {A_e},\ {B_e}} )$ is the similarity score of the two epigenomic profiles, and *w* is the epigenome weight. We adopt the TKF model (27) for the computation of $S( {\alpha ,\ {A_g},\ {B_g}} )$, while $E( {\alpha ,\ {A_e},\ {B_e}} )$ is computed using our recently developed genome-epigenome co-evolution Model I (17). In the alignment process, EpiAlignment scores each pair of nucleotide bases from two sequences, rewarding matches and penalizing mismatches and gaps. On top of aligned bases, EpiAlignment rewards matched epigenomic states (1-1 and 0-0) and penalizes mismatches (1-0 and 0-1) (Supplementary Figure S2). By integrating the sequence and epigenomic scores together, the algorithm maximizes the target function to identify the optimal alignment ${\alpha _{{\rm{opt}}}} = \ {\rm{arg}}\mathop {\max }\limits_\alpha T( {\alpha ,\ A,\ B} )$.

### Database for pairwise ChIP-seq experiments

The EpiAlignment web server contains a database of pairwise ChIP-seq experiments, which contains ChIP-seq datasets curated from the RoadMap Epigenomics Project (23), the ENCODE Project (24,25), the mouse ENCODE Project (6) and published epigenomic comparative studies (11). The current release of EpiAlignment includes 56 human datasets and 70 mouse datasets, obtained from 3 cell lines, 8 adult tissue types and 4 embryonic tissue types (Table 1). Human and mouse tissue types were matched by their bio-sample meta-data, including tissue/organ type, life stage and donor status.

**Table 1.** Number of ChIP-seq datasets in the database for pairwise ChIP-seq experiments

	H3K4me3		H3K27ac		H3K4me1	
Tissue/cell type	Human	Mouse	Human	Mouse	Human	Mouse
Adult B-lymphocytes	2	3	1	1	1	2
Adult erythroid cells	2	3	1	1		
Adult kidney	2	1	2	1	2	1
Adult liver	3	1	2	1	4	1
Adult spleen	2	1	3	1	3	1
Adult testis	1	1				
Adult cerebellum	1	1				
Adult lung	1	1			1	1
Adult small intestine	2	1	2	1	1	1
Adult round spermatids	1	2				
Embryonic stem cells	1	2	1	1	1	2
Embryonic heart	1	8			2	8
Embryonic kidney	1	3			1	3
Embryonic lung	2	3			3	3
Embryonic stomach	1	3	1	3	1	3

Rows: cell types or tissues of different life stages. Columns: datasets of different histone modifications, separated by species. Empty cells in the table represent absence of comparable datasets in the corresponding tissue/cell types.

ChIP-seq experiments for H3K4me3, H3K27ac and H3K4me1 were selected in the current release because of their prominent role in enhancer and promoter activities. For each ChIP-seq experiment, only the results containing stable pooled peak regions from multiple isogenic biological replicates and passing the quality control criteria of the consortia were included in the final pairwise database.

### Database for evolutionarily-related genes

EpiAlignment provides a database of evolutionarily-related genes, namely gene clusters, to assist users in functional comparison. The gene clusters were identified by grouping paralogous genes in each species and linking orthologous genes across species. The database contains a total of 2,607 pre-identified gene clusters, including 8,000 human genes and 10,211 mouse genes. Genes without annotated paralogues are not included in the database. The gene clusters are indexed by the gene names and aliases obtained from NCBI annotation database. When users provide a partial name or an Ensembl ID of the gene, EpiAlignment will list all clusters containing the gene(s) with a matching gene name, alias or Ensembl ID for users to choose from.

## WEB SERVER DESCRIPTION

### Overview

EpiAlignment provides two major alignment modes: the one-vs-one mode and the many-vs-many mode. The default one-vs-one mode attempts to find a best-matching part for a query region in its corresponding target region. It is useful in cases where target regions are much longer than query regions, and best matches of the query regions cannot be easily determined in the target species with sequence homologies only. The many-vs-many mode, on the other hand, is designed to identify the best match for a query region among a set of target regions. It is more useful when the query region corresponds to multiple candidates with potentially similar functions, such as promoters of paralogous genes.

### Input

EpiAlignment requires two types of inputs from users in both modes: (i) a pair of peak files from ChIP-seq experiments and (ii) query and target regions to be aligned, both from two species. With the genome assemblies specified, users can either upload their own peak files or select a pair of epigenomic datasets from the database of pairwise ChIP-seq experiments. Details of each ChIP-seq experiment can be viewed on the ENCODE or the GEO website by following the links provided in the database. The query and target regions can be provided in various ways depending on the alignment mode used (Table 2).

**Table 2.** Alignment modes and supported target region types

Alignment mode	Supported target region type	Description
One-vs-one mode	Homologous regions	For each query region, search for the best-matching sub-region in the neighborhood of its homologous region.
	Genomic coordinates	For each query region, search for the best-matching sub-region within a designated target region.
Many-vs-many mode	A gene cluster	Align each query region against all isoform promoters in a group of evolutionarily-related genes to identify the best-matching promoter.
	Genomic coordinates or gene names	Align each query region against all target regions to identify the best match.

#### Specifying query regions

In both modes, query regions can be provided as genomic coordinates in BED6 format. Gene symbols or Ensembl IDs are also accepted if gene promoters are used as input regions. In the latter case, users need to specify which range around gene transcription start sites (TSSs) will be defined as promoter regions. By default, the (–1000, +500) flanking regions around TSSs are used.

#### Specifying target regions

In the one-vs-one mode, target regions can be defined based on homologous relationship. Users only need to specify the number of base pairs by which the query regions will be increased. The web server will extend each query region and remap it to the target species. The remapped region will be used as the target region. Target regions can also be provided as a list of genomic coordinates in BED6 format. In this case, query and target regions have to be paired, with corresponding lines in the two lists specifying query-target region pairs.

In the many-vs-many mode, the target regions can be specified in the same way as query regions in the ‘Target regions’ box, or by selecting a group of evolutionarily-related genes. Users can search for a preset gene cluster by typing a gene symbol or an Ensembl ID in the searching bar. All gene promoters in the selected cluster will be used as target regions. The counts of query and target regions can be different in this mode as they will be aligned all-against-all.

With the query and target regions defined, the user may adjust the epigenome weight and alignment parameters (Supplementary Table S1). This step is optional as default parameters are provided on the webpage. Finally, the user can initiate the alignment by clicking the ‘Submit’ button.

### Selection of the epigenome weight

Users may adjust the epigenome weight (*w*) to leverage DNA sequence and epigenomic information in the alignment. The algorithm considers only sequence when *w* = 0, while it increasingly relies on epigenomic information as *w* rises.

A major consideration of selecting the weight is to what extent the epigenomic contribution entirely overrides the sequence contribution. Specifically, even with completely different epigenomic modification patterns, regions with highly conserved sequences are not expected to be aligned to *random* locations with consistent epigenomic patterns (random epigenomic peak). We simulated this case to study the effects of various weights (Supplementary Methods). We identified mouse genomic regions marked by H3K4me3, H3K27ac or H3K4me1 in all tissue types included in our pairwise ChIP-seq experiment database, among which around 50% had their human orthologues identified using liftOver (28). For each histone mark, 5,000 mouse regions were randomly selected as queries for the analysis, and their human orthologues were extended to define target regions. We assigned simulated epigenomic signals (‘1’s) to each mouse region, whereas no signal (‘0’s) to its human orthologous counterpart. We also assigned epigenomic signals (‘1’s) to a random location near the human orthologue, namely a ‘decoy’, to simulate a random peak within the human target region (Supplementary Figure S3A).

We first assessed the sequence similarities of each mouse query to its human orthologue and the decoy with sequence-only alignment (*w* = 0). We denoted the sequence alignment score (*S* score) between the mouse query and its human orthologue by *S*_ortho_, and the *S* score between the mouse query and the decoy as *S*_decoy_. The *S*_ortho_ distributions were similar among mouse sequences marked by the three histone modifications, with the first quartiles overlapping with the background distribution generated with random mouse and human sequences (Supplementary Figure S3B).

We then asked with what *w* value, the decoys would overtake the orthologues. Toward this goal, we ran EpiAlignment with *w* varying from 0.01 to 0.3. With each *w*, we defined a mouse query region as ‘misaligned’ if the decoy rather than the orthologue was identified as the best EpiAlignment hit. When *w* was close to 0, only the least conserved mouse regions were aligned to the decoys. As *w* increased, more conserved mouse regions with higher *S*_ortho_ scores started to be misaligned, and the differences between their *S*_ortho_ and *S*_decoy_ scores enlarged. When *w* exceeded 0.15, mouse regions with *S*_ortho_ scores above the medians began to be misaligned (Supplementary Figure S3C). Thus, *w* values larger than 0.15 are not recommended.

In real analysis, *w* also determines whether a location with lower sequence identity but higher epigenomic similarity will overtake the best sequence match when epigenomic information is incorporated. The user may increase *w* to include hits with lower sequence similarities, or decrease *w* to limit the results to hits with *S* scores comparable to the best sequence matches. For each alignment result, the contribution of sequence and epigenomic similarity can be further scrutinized with metrics provided on the result page to filter out hits undesired.

### Output

The user will be redirected to the result page after successfully submitting the data. The result page will refresh automatically until the task is done. A URL to the page will also be sent to the user if an email address is provided.

In both modes, EpiAlignment will return two types of alignment results, yielded with (*w* > 0) and without (*w* = 0) epigenomic information. For each query region, best matches identified in the two alignments are named ‘EpiAlignment hit’ and ‘Sequence-only hit’ respectively, with alignment scores denoted by ${T_{{\rm{Epi}}}},\ {T_{{\rm{Seq}}}}$ when *w* > 0, and ${S_{{\rm{Epi}}}},\ {S_{{\rm{Seq}}}}$ when *w* = 0. All scores are calibrated to 1,000 bp (i.e. alignment score per kilobase), and are thus comparable across alignment results of different region pairs. Alignment results are presented in an interactive table on the result page. Users may click on each row to open an expandable panel and view more details.

#### Output of the one-vs-one mode

In this mode, each expandable panel contains a chart presenting alignment scores along the target regions and a result evaluation panel, which consists of four parts:

- Overview: this part shows alignment scores of the two hits. A badge will be displayed if the coordinates of the two hits do not overlap, suggesting that the query region is aligned to another location after taking epigenomic information into account.
- Sequence evaluation: this part evaluates sequence similarities between the query region and the two hits. For every hit, a signal-to-noise ratio (SNR) is provided comparing its sequence alignment score to those of arbitrary locations within the target region (Supplementary Methods). An SNR no larger than 1 indicates that the hit's sequence similarity to the query is indistinguishable from random sequences nearby, and thus the sequence similarity scarcely contributes to the overall similarity at the location. Ranges of sequence alignment scores yielded with random sequences and mouse-human orthologous sequence pairs are also provided for reference (Supplementary Methods).
- Epigenome evaluation: this part evaluates the epigenomic similarity scores (*E*) between the query and the hits, defined as ${E_{{\rm{Epi}}}} = {T_{{\rm{Epi}}}}\ - {S_{{\rm{Epi}}}}$ and ${E_{{\rm{Seq}}}} = {T_{{\rm{Seq}}}}\ - {S_{{\rm{Seq}}}}$. A theoretical range of *E* is provided based on the query epigenomic profile, where the maximum and the minimum can be achieved when all epigenomic states are rewarded or penalized, respectively. Within the range, a larger *E* value suggests higher epigenomic similarity, and *vice versa*.
- Expression values of surrounding genes: this panel shows expression levels of genes (FPKM values) within 100 kb flanking regions of the query and target regions.

#### Output of the many-vs-many mode

In this mode, the expandable panels contain sequence alignment score ranges yielded with random and orthologous human-mouse promoter pairs for the evaluation of sequence similarities (Supplementary Methods). Beside the table, two heatmaps will be returned illustrating alignment scores between query regions (rows) and target regions (columns). For each query region, the EpiAlignment hit will be considered as ‘altered’ from the Sequence-only hit if the two hits are different regions, and the epigenomic profile contributes positively within the EpiAlignment hit (${T_{{\rm{Epi}}}} >{S_{{\rm{Epi}}}}$) but negatively within the Sequence-only hit (${T_{{\rm{Seq}}}} < {S_{{\rm{Seq}}}}$). In this case, the EpiAlignment and Sequence-only hits are highlighted by green and gray boxes respectively. If input regions are defined with gene symbols or IDs, the gene expression levels will be provided in FPKM values and presented in a bar chart next to the heatmaps when the data are available.

In both modes, all genomic regions can be viewed individually in the UCSC genome browser by clicking the icon beside each genomic coordinate, or in region pairs in GIVE by clicking the icon at the beginning of the rows. Users may select ChIP-seq data from custom tracks and navigate in the browsers to further investigate genomic contexts of the regions.

### Runtime and computational performance

The time complexity of the algorithm is O(*mn*) where *m* and *n* are the lengths of regions to be aligned. The architecture of EpiAlignment utilizes parallel computing of up to 140 worker threads. A load-balancer powered by Nginx is set up at the front of the server to route requests from multiple users to an appropriate state-less Node.js handler, which can allocate region pairs among available worker threads to achieve parallel computing.

We performed a simulation test of concurrent jobs running on the EpiAlignment server in the default mode with different job sizes (10 and 20 region pairs per job, with 2,000 bp query regions and 20,000 bp target regions). For both sizes, a single job took about 1.25 minutes to finish. The computational resources on the server were not saturated for small-sized jobs and the runtime were merely increased to 2 minutes with 10 concurrent jobs. When the job size doubled, EpiAlignment showed resource saturation at five concurrent jobs and the runtime increased moderately to 4 minutes for 10 jobs running simultaneously (Supplementary Figure S4). In all cases, the workload was balanced among available workers, yielding nearly the same runtime for all concurrent jobs.

## DATA APPLICATIONS

### EpiAlignment recapitulates previously identified regulatory sequences

We built the EpiAlignment web server to formalize the previously validated comparative epigenomics method (7) and make this method accessible to researchers at large. Our previous analysis revealed that the genomic sequences with moderate interspecies sequence conservation and conserved H3K27ac histone modification often exhibited enhancer activities (7). The analysis relied on an *ad hoc* method, which was subsequently improved into a probabilistic model (17). This probabilistic model serves as the theoretical foundation of the target function used in EpiAlignment.

We tested whether EpiAlignment could recapitulate the main finding of our previous analysis. Back in 2011, other work reported lack of correlation between the temporal histone modification changes on promoters and the temporal changes of gene expression in a differentiation process (29). Thus, it was not trivial to identify putative regulatory sequences that exhibited correlated temporal histone modification changes with the expression changes of their nearby genes (7). In this test, we aligned all 76,094 human H3K27ac-marked regions in embryonic stem (ES) cells to the mouse genome with H3K27ac ChIP-seq data of human and mouse ES cells. 9,418 human H3K27ac peaks were aligned to mouse regions marked by H3K27ac, among which the 4,015 hits with SNR larger than 1.5 were kept for downstream analysis. Of the 4,015 mouse regions, 64% retained the H3K27ac peaks through cell differentiation day 6 (1→1), 33% lost the H3K27ac peak during cell differentiation (1→0), and the rest 3% showed non-monotonic H3K27ac trends. The peaks were then assigned to the nearest genes within their 50kb surrounding regions. The genes with nearby conserved H3K27ac peaks in undifferentiated cells exhibited an overall decrease of expression during the differentiation process (blue and red lines, Supplementary Figure S5). However, the genes near the conversed H3K27ac peaks in undifferentiated cells that lost H3K27ac during differentiation (1→0, red line) exhibited greater decrease of expression than those that did not lose H3K27ac during differentiation (1→1, blue line, Supplementary Figure S5). Thus, EpiAlignment identified the genomic sequences that exhibited correlated histone modification changes with the expression changes of their nearby genes. EpiAlignment recapitulated the main results of our previous paper (7).

### Case studies

Two examples with sample inputs were provided on the EpiAlignment website intended to illustrate the usage of the two alignment modes.

#### Case study 1

**Figure 2.**
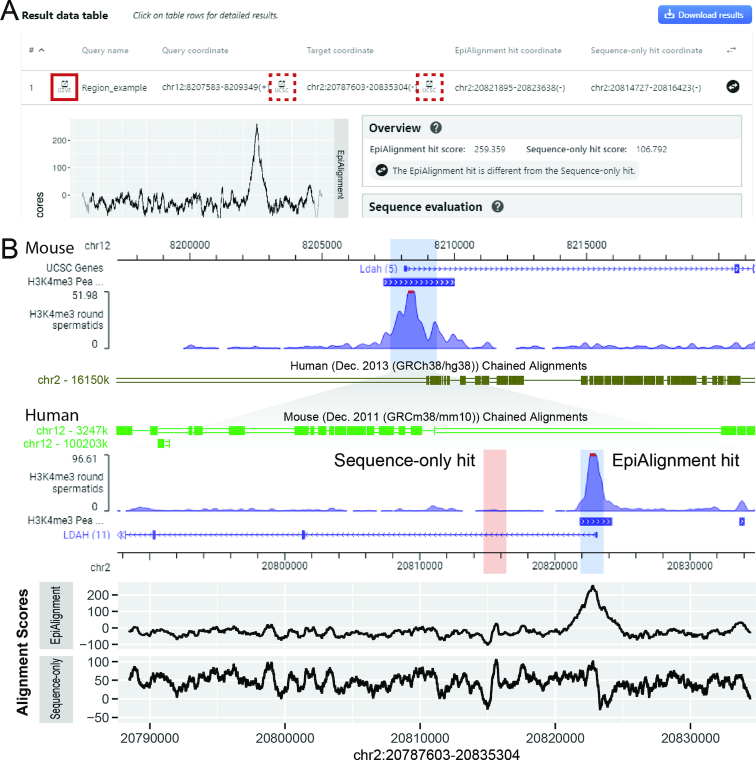
One-vs-one mode results. (**A**) The interactive table containing coordinates of input regions and best matches. Users can click on each row to expand it and view the result evaluation panel. Users may also visualize the query and target regions together in GIVE by clicking the icon at the beginning of each row (solid red box), or individually in UCSC Genome Browser by clicking the icon behind each coordinate (dotted red box). (**B**) Visualization of the query and target regions in GIVE, together with alignment scores along the target region. Upper: screenshot of GIVE showing the query region (blue shaded area at top) as well as the EpiAlignment hit (blue shaded area) and the Sequence-only hit (pink shaded area) within the target region (bottom). Human and mouse chained alignments are added to demonstrate the sequence alignability across the two species. Lower: alignment scores along the target region, produced by EpiAlignment (top) and sequence-only alignment (bottom). X-axis: genomic coordinates aligned to the GIVE view. Y-axis: alignment scores.

This case study demonstrates how to find regions with similar chromosomal structures using the default mode. The promoter regions of human gene *LDAH* and its orthologous mouse gene *Ldah* are both marked by H3K4me3 in round spermatids, whereas the underlying sequences are poorly conserved. With liftOver (28), a sequence homology-based tool, the mouse promoter was mapped to a human intronic region ∼10 kb from human *LDAH* promoter, and the result human region showed no H3K4me3 occupancy. We used the H3K4me3-marked region in mouse *Ldah*’s promoter as the input query region (chr12:8,207,583–8,209,349) and aligned it against the –20,000 to +20,000 neighborhood of its orthologous region in human. The H3K4me3 ChIP-seq datasets of human and mouse round spermatids were selected from the database (GEO accession ID: GSM1673960 and GSM1674016) as epigenomic information (11). The sequence-only alignment (*w* = 0) revealed two hits with similar alignment scores. The downstream one corresponded to *LDAH*’s promoter, while it showed an alignment score slightly lower than the best sequence match. With the H3K4me3 information, the best hit shifted to *LDAH*’s promoter, and EpiAlignment successfully matched the two promoter regions together (Figure 2). Given the orthologous relationship between the two genes, it is more reasonable that their promoters share similar functions.

We repeated the analyses with the same query and target regions using human and mouse testis H3K4me3 ChIP-seq data (ENCODE accession ID: ENCSR611DJQ and ENCSR000CCW). EpiAlignment identified the corresponding promoter pairs (blue shaded area, Supplementary Figure S6A), whereas sequence-only alignment misaligned the mouse query to an intronic sequence (pink shaded area). Furthermore, we repeated this analysis with human and mouse testis H3K27ac ChIP-seq data (ENCSR136ZQZ and ENCSR000CCU). Again, EpiAlignment correctly aligned the promoter sequences whereas sequence-only alignment failed to align them (Supplementary Figure S6B). This case illustrated how EpiAlignment could be used to identify functional genomic sites with both sequence and epigenomic similarities in regions lacking sequence conservation.

#### Case study 2

We asked whether best matches of promoter pairs derived by EpiAlignment and sequence-only alignment always agreed. If they did not agree, which promoter alignment would be consistent with orthologous gene pairs. Toward this goal, we aligned the promoter of the human *SLCO4A1* gene against the promoters of mouse genes within the solute carrier organic anion transporter family using the many-vs-many mode. The H3K4me3 ChIP-seq datasets of human and mouse B-lymphocytes (ENCSR057BWO and ENCSR000CGK) were selected as the epigenomic input data. We used the gene symbol *SLCO4A1* to define the query region. In the ‘Target regions’ box, we selected ‘Search a gene cluster’ and typed ‘SLCO4A1’ to search for a gene cluster containing its homologous genes. The resulting ‘Cluster_6635’ was selected from the pop-up panel as input. This cluster contained 16 mouse genes, among which *Slco4a1* was the orthologue of *SLCO4A1*.

**Figure 3.**
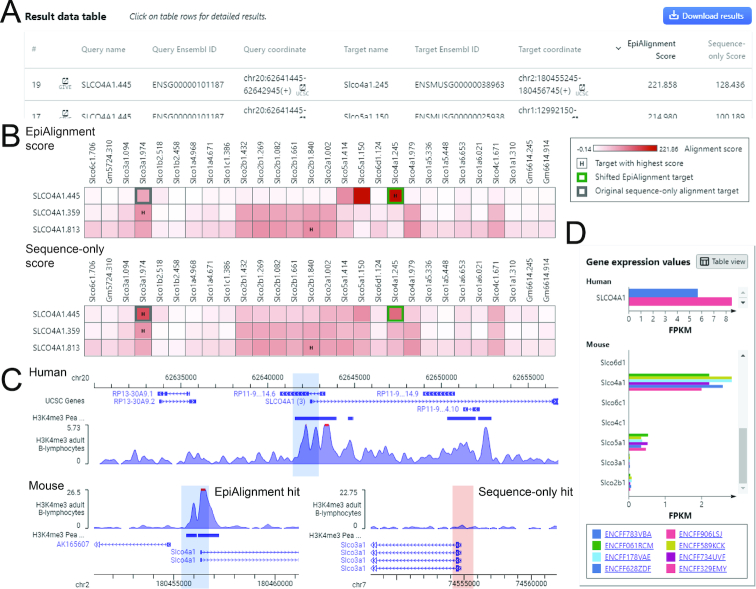
Many-vs-many mode results. (**A**) The interactive table containing coordinates of input regions and best matches, alignment scores and links for visualization. The user may sort the table by each column by clicking on the column name. (**B**) Heatmaps of alignment scores on the result page. Rows: query regions. Columns: target regions. Color-scale in the cells: alignment scores of query-target region pairs. Green box: the best match identified by EpiAlignment. Gray box: the best match identified by sequence-only alignment. ‘H’ in the cell: the target region with the highest alignment score in the row. (**C**) Visualization of the regions in GIVE. Top: the genomic context of the input query region (blue shaded area). Bottom: the genomic context of the best matches found by EpiAlignment (left, blue shaded area) and by sequence-only alignment (right, pink shaded area). (**D**) A bar chart of expression values of the query and target genes on the result page.

With sequence data alone, *SLCO4A1′*s promoter was aligned to *Slco3a1′*s promoter instead of its orthologue, whereas after incorporating the H3K4me3 data, the promoters of *SLCO4A1* and *Slco4a1* were successfully matched (Figure 3A, B). The alignment score between the orthologous promoters increased after taking epigenomic data into account, suggesting consistent H3K4me3 occupancies within them. The visualization of results showed that *SLCO4A1′*s and *Slco4a1′s* promoters both harbored H3K4me3 peaks, whereas *Slco3a1′*s promoter exhibited little H3K4me3 signals (Figure 3C). Moreover, *SLCO4A1* and *Slco4a1* were both expressed in B-lymphocytes In line with their orthologous relationship, whereas all other mouse genes exhibited no or little expression (FPKM < 1) (Figure 3D). This example presented a case where promoters of orthologous genes could be paired using EpiAlignment, but not with sequence data only.

## DISCUSSION

Integrative analyses of the genome and the epigenome can provide insights for the annotation of regulatory sequence, especially in regions lacking sequence conservation. We have developed EpiAlignment, a cross-species alignment tool incorporating both the genome and the epigenome. By assessing structural similarities between functional genomic sites, the tool may assist researchers in identifying functional correspondence across species, which provides a starting point for downstream experiments and analyses. The interface of EpiAlignment is designed to be user-friendly and does not require users to be experienced in computational biology. It also shows versatility in providing different alignment modes and supporting various input types. Users may easily use the one-vs-one mode on orthologous neighborhoods of regulatory elements to refine their annotations, or search for a pre-defined gene cluster and align them with the many-vs-many mode to study the potential functional correspondence among evolutionarily-related genes.

Given that EpiAlignment uses peak files from epigenomic experiments to assess epigenomic signatures within regions, its output can be affected by the peak-calling results. Therefore, we only included stable peak files from ENCODE experiments with isogenic biological replicates pooled in our database for pairwise ChIP-seq experiments. When using custom data, users may adjust thresholds for peak-calling and examine the genomic regions surrounding the peaks in genome browsers for better performance. Peak significance may be incorporated into the target function in the future to improve the robustness of the results.

EpiAlignment can be further enhanced in several aspects in the future. First, the database for pairwise ChIP-seq experiments can be expanded to include more comparable datasets of different experiment types, including DNase-Seq, ATAC-Seq and MeDIP-Seq. Species other than human or mouse can also be supported in future updates when sufficient epigenomic data are available. Further expansion of the algorithm may allow EpiAlignment to align with multiple epigenomic marks simultaneously to better reflect the interactions among different epigenomic modifications. Moreover, while the current release of EpiAlignment does not support genome-wide alignments due to speed constraints, future investments in computational hardware and algorithm developments may improve the speed performance and enable alignments against the entire genome.

## Supplementary Material

gkz426_Supplemental_Files
