# Supplementary material for: EpiAlignment: alignment with both DNA sequence and epigenomic data
Source: Nucleic Acids Res. 2019 May 22;47(W1):W11–9. doi: 10.1093/nar/gkz426 (PMC6602515; doi:10.1093/nar/gkz426)
Supplement: gkz426_Supplemental_Files [file gkz426_supplemental_files.pdf]

SUPPLEMENTARY METHODS

Generating simulation data for weight selection.

Mouse regions marked by H3K4me3, H3K27ac or H3K4me1 were first identified in all tissue types included in the pairwise ChIP-Seq experiment database of the web server. The identified mouse regions were merged by histone modifications, split into fragments no longer than 2,000 bp to generate potential genomic functional sites in mouse. These regions were then remapped to the human genome using liftOver with minMatch = 0.5 to find their human orthologues. For each of the three histone marks, 5,000 mouse-human orthologous region pairs were randomly selected as query regions for downstream analyses. All human regions were extended to 4-fold of their original lengths by random distances upstream and downstream to generate target regions for alignment. Within each region pair, simulated epigenomic signals ("1"s) were assigned to all bases in the mouse query region. Epigenomic signals ("1"s) were also assigned to a random location with the same length within the human target region, whereas the rest of the human region were assigned with "0"s. The mouse and human regions were aligned with weights varying from 0 to 0.1 with step length 0.01, and from 0.15 to 0.3 with step length 0.05. The sequence-only alignment (w = 0) was repeated with three sets of randomly selected region pairs to make sure that the results were insensitive to random sampling.

Besides, 15,000 mouse regions were randomly selected from the mouse genomic functional sites and aligned to 6kb-long human regions randomly selected from the entire genome. The averaged sequence alignment scores along the target regions were then used to generate the background distribution.

Calculating signal-to-noise ratio (SNR)

After alignment, the target region is divided into 500bp bins, within each of which the maximum and minimum of sequence-only alignment scores are computed. The 25th percentile of the minima and 75th percentile of the maxima are used as the lower and upper bound of background noise, denoted by l and u respectively. Let S denotes the sequence-only alignment score between the query region and a hit, the hit's SNR is then computed as the following:

$$SNR = \frac{2S - (u + l)}{u - l}$$

Generating reference distributions for sequence evaluation

In both modes, two sequence-only alignment score (S score) distributions are provided within the expandable for users' reference.

In the one-vs-one mode, the background distribution is identical with that generated in the simulation test (see "Generating simulation data for weight selection"), whereas the S score distribution of conserved mouse-human region pairs was generated with all mouse-human orthologous region pairs (approximately 15,000 pairs) used in the simulation test, regardless of histone modifications.

In the many-vs-many mode, the background distribution was generated by aligning 10,000 pairs of randomly-paired mouse and human promoters. The S score distribution of orthologous mouse-human promoter pairs was generated using all promoter pairs whose corresponding human and mouse

genes are unique orthologues to each other. Only genes included in the database for evolutionarily-related genes were used (4,685 pairs in total).

SUPPLEMENTARY FIGURES AND TABLES

Supplementary Figure 1. Demonstration of the many-vs-many mode. Left: query and target regions are provided as inputs by the user, together with the epigenomic data (purple). Sequence similarities vary among the query and target regions (grayscale in bars). Each query region is aligned against all target regions (dashed double-headed arrows). Right: The EpiAlignment output. The alignment score is shown in red color for each pair of query region (row) and target region (column).

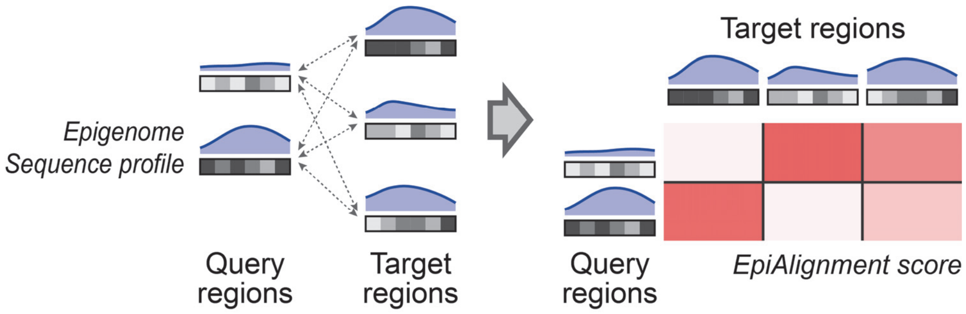

Supplementary Figure 2. Demonstration of data types and scoring scheme. An alignment between a pair of DNA sequences (A_g, B_g) with epigenomic profiles on top of them (A_e, B_e) is shown. Indels, matches and mismatches in the sequence alignment are represented by −, | (black) and | (red), respectively. Peak regions of the epigenomic modification are shown as blue bands. The epigenomic profiles, A_e and B_e, are represented by binary values on each base, with 1 being inside a peak and 0 being outside the peaks. Insert: scoring scheme for epigenomic states on aligned bases, with matched states being rewarded (green) and mismatched states being penalized (red).

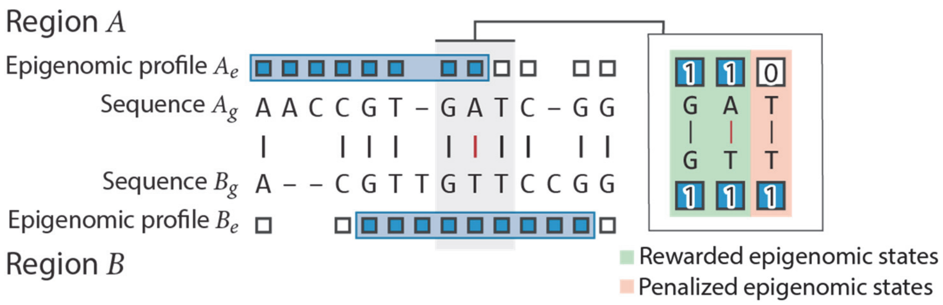

Supplementary Figure 3. Simulation tests for epigenome weight selection. (A) Structure of a region pair generated in the simulation test. Red boxes: a selected mouse region (above, used as the query region) and its human orthologous region (below), with the red shading showing the orthologous relationship between them. Gray band: the flanking region of the human orthologue, used as the target region. Blue bars: simulated epigenomic signals assigned to the mouse query region and a random region other than the human orthologue, namely a “decoy” (dotted). (B) Distributions of sequence alignment scores (S score) between mouse regions marked by H3K4me3, H3K27ac or H3K4me1 and their human orthologues (S_{ortho} score) as well as random human genomic regions. (C) Sequence-only alignment scores S_{ortho} and S_{decoy} of misaligned mouse regions under different weights (w). Each w corresponds to an individual set of misaligned mouse regions. The distributions of S_{ortho} (red) and S_{decoy} (blue) scores of the misaligned regions are plotted on y-axis. Solid colored lines represent medians of the S scores, and shaded areas represent the ranges of S scores between the 10th and 90th percentiles. S scores of all mouse-human orthologous region pairs (black) and random region pairs (gray) are plotted for reference, with solid lines and dotted lines representing the medians, the 25th and the 75th percentiles of the S scores, respectively.

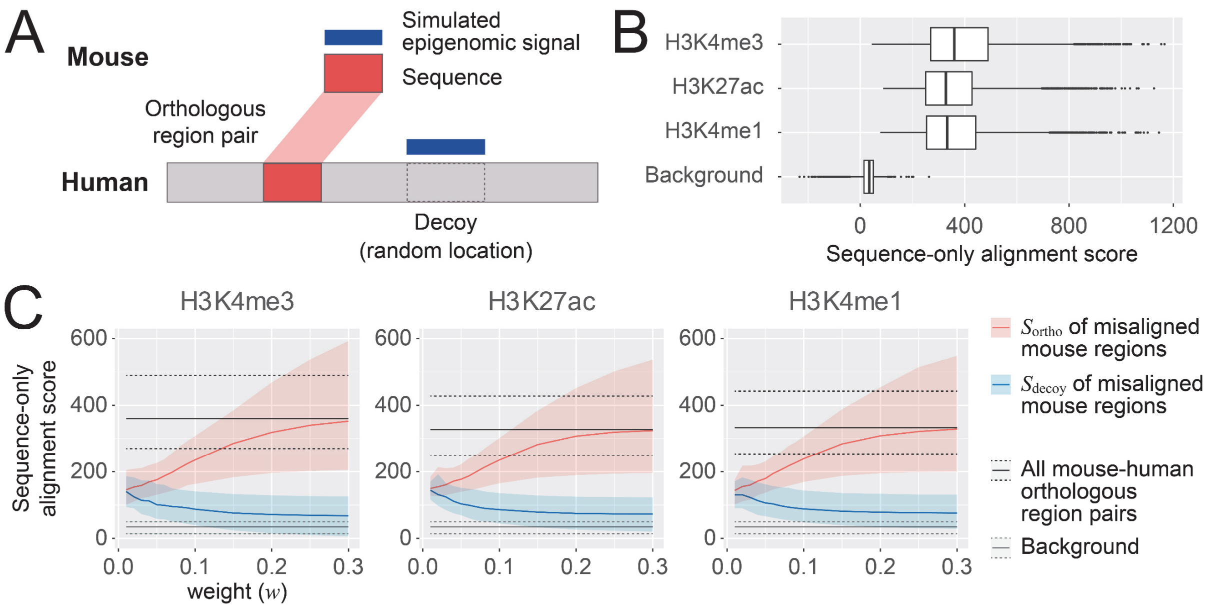

Supplementary Figure 4. Web server runtime analysis. Runtime (y-axis) of EpiAlignment with different numbers of concurrent jobs (x-axis) and job sizes (blue and orange). Each dot represents the runtime of one job run.

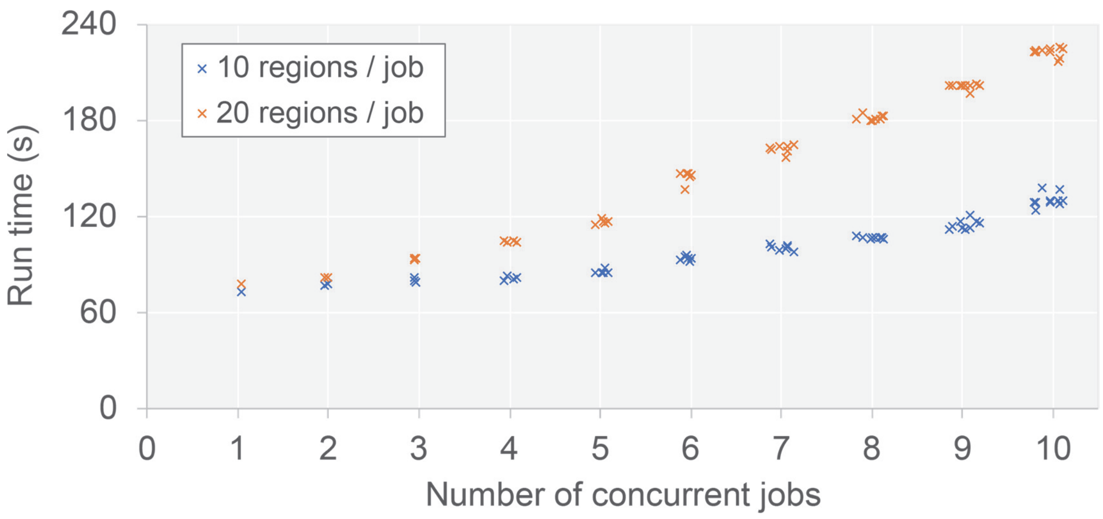

Supplementary Figure 5. H3K27ac changes and gene expression changes during embryonic stem (ES) cell differentiation. The lines represent log expression changes of genes associated with a set of mouse regions identified by EpiAlignment. All mouse regions are marked by H3K27ac in undifferentiated mouse ES cells on day 0, and are categorized into two classes: regions with retained H3K27ac during ES cell differentiation (1→1), and regions with lost H3K27ac (1→0). Log expression values of their nearest genes on cell differentiation day 4 and day 6 are normalized against day 0 and plotted on y-axis. The error bars represent standard errors of the mean.

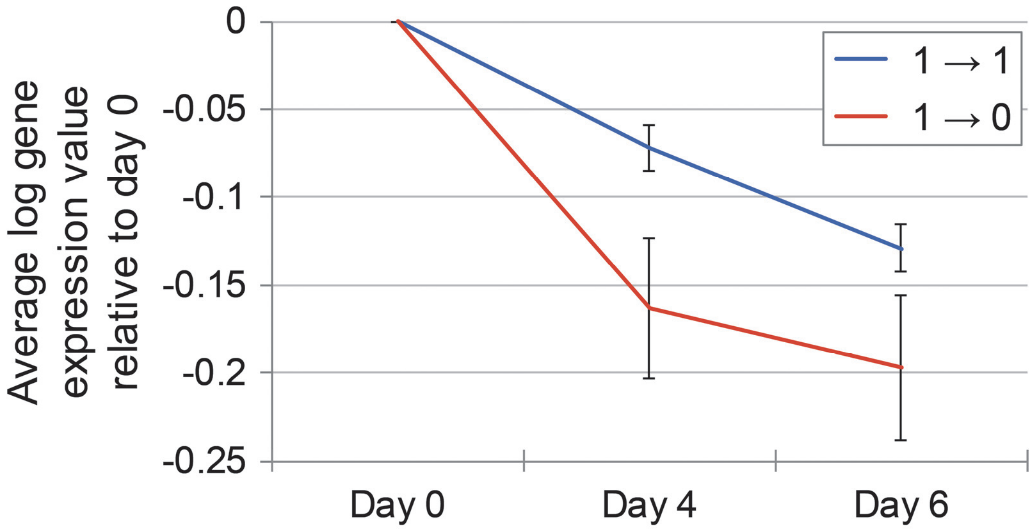

Supplementary Figure 6. Alignment results of the mouse gene *Ldah*'s promoter using epigenomic data of adult testis. Result tables and GIVE browser views of the alignments using ChIP-Seq data of (A) H3K4me3 and (B) H3K27ac in adult testis from the ENCODE Project.

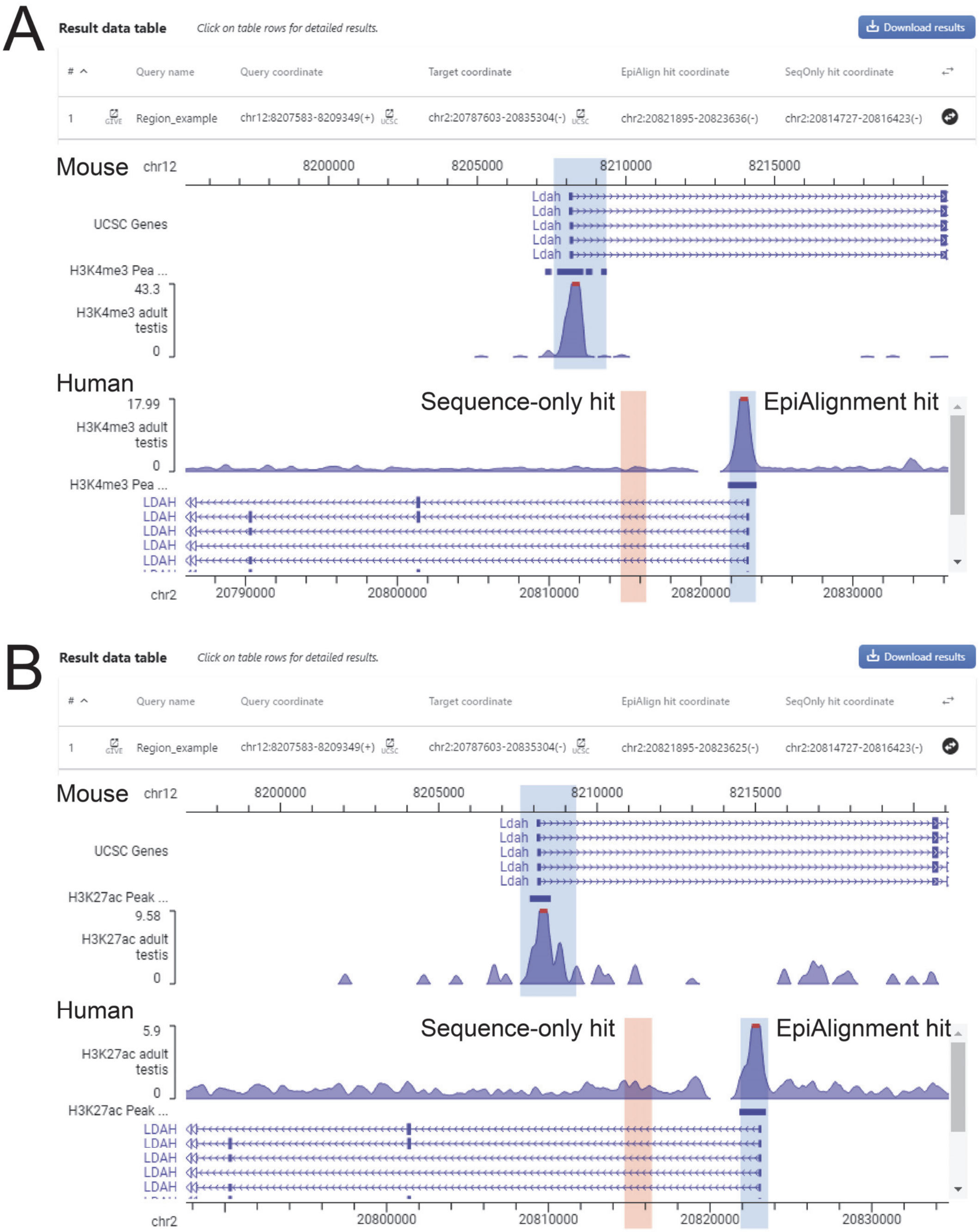

Supplementary Table 1. EpiAlignment parameters.

Parameter	Description	Theoretical range	Default value
w	Epigenome weight. The epigenome alignment score is multiplied by *w* in the target function.	$(0, +\infty)$	0.1
s	DNA base substitution rate. With a smaller *s*, larger reward and penalty scores will be assigned to matched and mismatched DNA bases, respectively.	$(0, +\infty)$	0.3
μ	DNA base deletion rate. With a smaller μ, a larger penalty will be assigned to insertions and deletions.	$(0, +\infty)$	0.3
κ	Switching rate between the binary epigenomic states 0 and 1. With a smaller κ, larger reward and penalty scores will be assigned to matched and mismatched epigenomic states, respectively.	$(0, +\infty)$	0.5
$\pi_A, \pi_C, \pi_G, \pi_T$	Equilibrium probabilities of the four nucleotide bases.	(0, 1)	0.25
π_1	Equilibrium probability of the epigenomic state "1", i.e. the probability of having epigenomic state "1" on a genomic location.	(0, 1)	0.1
